# Postoperative hormonal therapy prevents recovery of neurological damage after surgery in patients with breast cancer

**DOI:** 10.1038/srep34671

**Published:** 2016-10-06

**Authors:** Atsushi Sekiguchi, Chiho Sato, Izumi Matsudaira, Yuka Kotozaki, Rui Nouchi, Hikaru Takeuchi, Masaaki Kawai, Hiroshi Tada, Takanori Ishida, Yasuyuki Taki, Noriaki Ohuchi, Ryuta Kawashima

**Affiliations:** 1 Department of Adult Mental Health, National Institute of Mental Health, National Center of Neurology and Psychiatry, Kodaira, Tokyo, Japan; 2 Division of Medical Neuroimage Analysis, Department of Community Medical Supports, Tohoku Medical Megabank Organization, Tohoku University, Sendai, Japan; 3 Department of Functional Brain Imaging, Institute of Development, Aging and Cancer (IDAC), Tohoku University, Sendai, Japan; 4 Division of Surgical Oncology, Graduate School of Medicine, Tohoku University, Sendai, Japan; 5 Department of Nuclear Medicine and Radiology, IDAC, Tohoku University, Sendai, Japan; 6 Division of Clinical Research, Medical-Industry Translational Research Center, Fukushima Medical University School of Medicine, Fukushima, Japan; 7 Creative Interdisciplinary Research Division, Frontier Research Institute for Interdisciplinary Science, Tohoku University, Sendai, Japan; 8 Human and Social Response Research Division, International Research Institute of Disaster Science, Tohoku University, Sendai, Japan; 9 Department of Advanced Brain Science, Smart Ageing International Research Center, IDAC, Tohoku University, Sendai, Japan; 10 Division of Developmental Cognitive Neuroscience, IDAC, Tohoku University, Sendai, Japan; 11 Department of Breast Oncology, Miyagi Cancer Center Hospital, Miyagi, Japan

## Abstract

Cancer survivors are exposed to several risk factors for cognitive dysfunction, such as general anesthesia, surgical trauma, and adjuvant therapies. In our recent study we showed that thalamic volume reduction and attentional dysfunction occurred shortly after surgery. Here, we examined the 6-month prognosis of the 20 patients with breast cancer who underwent surgery. Seven patients did not receive any adjuvant therapy after the surgery and 13 patients received a hormonal therapy after the surgery. We assessed their attentional functions, and thalamic volumes shortly after and 6 months after surgery. We found a significant group x time interaction in the attentional functions (p = 0.033) and the right thalamus (p <  0.05, small volume correction), suggesting the thalamic volume reduction and attentional dysfunction recovered in patients without adjuvant therapy. Our findings provide a better understanding of the potential role of hormonal therapy in relation to the cognitive dysfunction of cancer survivors.

Cancer survivors are exposed to several risk factors for cognitive dysfunction, such as general anesthesia, surgical trauma, the occurrence of complications, and adjuvant therapies^1^,^2^,^3^,^4^,^5^. The cognitive dysfunction after surgery is known as postoperative cognitive dysfunction (POCD)^1^,^2^,^3^. General anesthesia and inflammation due to surgical trauma or complications are considered the principal causes of POCD^6^,^7^,^8^. Similarly, the cognitive dysfunction caused by chemotherapy is a side effect of cancer therapy. Many anticancer drugs have neurotoxic effects, which cause cognitive dysfunction and brain atrophy^9^ termed “Chemo-brain”^4^,^10^.

The maintenance of cognitive function is important to preserve the quality of life (QOL) for breast cancer survivors^11^,^12^, because of the long-term survivorship. Previous studies have reported that cognitive dysfunction in patients with breast cancer may originate in neuron injuries caused by chemotherapy^13^, represented by abnormalities of specific brain structures^14^,^15^ and brain functions^16^,^17^. Similarly, cognitive functions were reported impaired by hormonal therapy^5^,^18^ and improved after the cessation of treatment^19^. However, a neuropathology of the cognitive dysfunction due to hormonal therapy remains unclear.

In our recent study, we investigated the cognitive dysfunction and brain structural changes shortly after surgery in patients with breast cancer and found thalamic volume reduction and attentional dysfunction were early responses to general anesthesia^20^. The results suggest the early postoperative response to anesthesia may represent an intermediate phenotype of POCD. Based on the previous findings of high incidence of POCD shortly after surgery, and rapid attenuation of symptoms as time progressed^2^, we assumed patients who underwent surgery with general anesthesia were similarly affected by the anesthesia and that a majority of these patients would fully recover after surgery if not exposed to other risk factors. Therefore, further investigations to distinguish the effects of hormonal therapy on brain structural changes and cognitive dysfunction are necessary.

To address this issue, we investigated these patients 6 months after surgery to clarify the prognosis of thalamic volume reduction and attentional dysfunction. To examine the effect of hormonal therapy, we controlled the risk factors for cognitive dysfunction after the surgery other than hormonal therapy. We hypothesized thalamic volume reduction and attentional dysfunction would recover in patients without hormonal therapy and not in those with hormonal therapy.

## Materials and Methods

### Participants

Thirty-two patients from our previous study who underwent breast cancer surgery^20^ were screened for eligibility in the current follow-up study. To control the effects of chemotherapy on cognitive function and brain, four patients who received chemotherapy after the surgery were excluded. Furthermore, to control the effects of serum estrogen levels on the brain, three patients who received hormonal therapy with a selective estrogen receptor modulator (SERM) such as tamoxifen were excluded, because SERMs do not decrease serum estrogen levels, whereas an aromatase inhibitor (AI) does. Five patients declined to participate in this study because they were either uninterested, or too busy. Finally, 20 postmenopausal women with early-stage breast cancer were enrolled in the current follow-up study. Seven patients did not receive any adjuvant therapy after the surgery (age = 51–62 yrs.; Wo group), while 13 patients received hormonal therapy with an AI after the surgery (age = 54–77 yrs.; Ho group). Demographic characteristics of patients are shown in Table 1. Written informed consent was obtained from each subject. The Ethics Committee of the Tohoku University Graduate School of Medicine approved this study. The methods of this study were also carried out in accordance with the approved guidelines.

### Protocol

Postoperative assessments were performed during hospitalization and follow-up assessments were performed when the participants came to the hospital as outpatients. Postoperative assessments were performed within 1 week of surgery (Post), and follow-up assessments were performed approximately 6 months after the surgery (Follow-up). Because the present study was observational in design, there were no restrictions on the types of anesthesia used. Assessments were also performed shortly before the surgery (for details, see^20^). The study was registered in the University Hospital Medical Information Network (UMIN) Clinical Trial Registry (UMIN 000007287).

### Psychological measurements

To evaluate the effect of adjuvant hormonal therapy, we assessed attentional function using the Digit Cancellation Test (D-CAT), which has been well validated in a Japanese population^21^. Given that we identified attentional dysfunction shortly after surgery using the D-CAT1 score in our previous investigation^20^, we focused on this score in the present follow-up study. The test sheet consisted of 12 rows of 50 digits, each containing five sets of numbers from 0–9 arranged randomly. Participants were instructed to identify the target number (6) with a slash mark as quickly and accurately as possible within 1 min. Based on the data from a Japanese population of each sex and age range^21^, we regarded scores < mean − 1 standard deviation in each age range (50 s: <23, 60 s: <19, 70 s: <16) as impaired.

### Image acquisition

All MRI data were acquired using a 3-T Philips Intera Achieva scanner (Best, Netherlands). A magnetization-prepared rapid gradient echo (MPRAGE) sequence was used to collect high-resolution T1-weighted structural images (240 × 240 matrix; 6.5-ms repetition time; 3-ms echo time; 24-cm field of view; 162 slices; 1.0-mm slice thickness).

### Voxel-based morphometry (VBM) analysis

Voxel-based morphometry (VBM) was performed to investigate persistent structural differences in patients treated with and without hormonal therapy after breast cancer surgery. The structural data were preprocessed using statistical parametric mapping software (SPM8; Wellcome Department of Cognitive Neurology, London, UK) implemented in Matlab (Mathworks Inc., Natick, MA, USA).

First, T1-weighted structural images of each individual were segmented into six tissue sections using the new segmentation algorithm implemented in SPM8. Default parameters were used for this new segmentation process, except for affine regularization, which was performed using the International Consortium for Brain Mapping (ICBM) template for East Asian brains. Then, we performed the DARTEL registration process implemented in SPM8. During this process, we used DARTEL-imported images of the two-tissue probability map (TPM) of gray and white matter, created using the abovementioned segmentation process. First, the template for the DARTEL procedure was created using T1WI data from the post-scan of all participants in the study. Next, we used this template to perform DARTEL (using default parameters) for the T1WI post- and follow-up scans of all subjects. The resulting images were then normalized spatially to the Montreal Neurological Institute (MNI) space to obtain images with 1.5 × 1.5 × 1.5-mm^3^ voxels. Additionally, we performed a volume change correction (modulation) by modulating each voxel with the Jacobian determinants derived from the spatial normalization, allowing regional differences in the absolute amount of brain tissue to be determined^22^. Subsequently, all images were smoothed by convolution with an 8-mm isotropic Gaussian kernel full-width at half-maximum (FWHM).

### Statistical analyses

Demographic differences for continuous variables (such as age) between the Ho and Wo groups were calculated using two sample *t-*tests. To examine the change of D-CAT1 scores 6 months after the surgery, we compared the Ho group with the Wo group using analysis of covariance (ANCOVA) models, after confirming that variance and distribution in the two groups were equal (p = 0.243) and normal (p = 0.200, in both groups) using the Levence test and Kolmogorov-Smirnov test, respectively. We used the follow-up scores as the dependent variables, the groups (Ho, Wo) as the categorical variables, and the post-score and age as covariates. A *p*-value <0.05 (one tailed) was considered to indicate statistical significance based on our hypothesis that attentional function would improve in the Wo group rather than the Ho group. All statistical analyses were performed using SPSS 18 (SPSS, Chicago, IL, USA).

Differences in regional gray matter volume (rGMV) were assessed as a group (Ho/Wo) x time (Post/Follow-up) interaction using an analysis of covariance (ANCOVA) model on SPM8. The analyses were performed using age, total brain volumes and the right thalamic volumes at post as covariates. Small volume correction (SVC) was applied to one region of interest (ROI) based on our previous findings (right thalamus)^20^ using anatomical masks from the “Human aal atlas” within the Wake Forest University PickAtlas 3.04 (http://fmri.wfubmc.edu/software/PickAtlas)^23^,^24^. A significance level was set at p = 0.05 corrected for multiple comparisons (voxel-level family-wise error). In the analyses, we included only voxels that showed GMV values >0.10 to avoid possible partial volume effects around the borders between grey and white matter, as well as between grey matter and cerebrospinal fluid. We also performed *post hoc* paired *t*-test on the right thalamic volumes for both groups at both visits, to verify the direction of the changes.

We performed a correlation analysis using the change in the ratio of the D-CAT1 score and right thalamic volume, which was obtained by dividing the post and follow-up scores and volumes by the post scores and volumes, respectively, to verify the relationship between changes in attentional function and thalamic volume.

## Results

**Figure 1:**
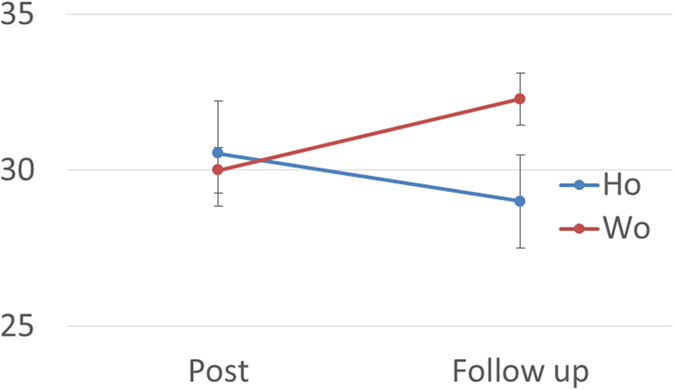
A significant group (Ho/Wo) × time (Post/Follow-up) interaction in the D-CAT1 scores (Wo: 30.0 ± 1.9 to 32.3 ± 2.2, Ho: 30.5 ± 6.1 to 29.0 ± 5.4, p = 0.033). The results showed the D-CAT1 scores in the Wo group improved, but not in the Ho group. Abbreviations: D-CAT, Digit Cancellation Task; Ho, hormonal therapy; Wo, without adjuvant therapy; Post, shortly after surgery; Follow-up, 6 months after surgery.

We found a significant group × time interaction in the D-CAT1 scores (Wo: 30.0 ± 1.9 to 32.3 ± 2.2, Ho: 30.5 ± 6.1 to 29.0 ± 5.4, p = 0.033, Fig. 1). Although the scores were estimated within normal ranges^21^ and attentional dysfunction was relatively mild, the results showed that attentional function improved in the Wo group but not in the Ho group over time.

**Figure 2:**
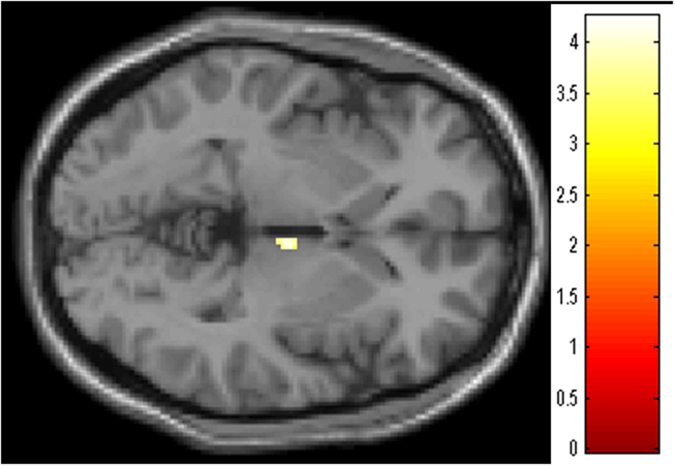
Regions showing significant group (Ho/Wo) × time (Post/Follow-up) interactions (right thalamus; x = 6, y = −12, z = −2; p < 0.05, svc). The colored bar shows the scale of the t-value. Abbreviations: Rt, right; Ho, hormonal therapy; Wo, without adjuvant therapy; Post, shortly after surgery; Follow-up, 6 months after surgery; svc, small volume correction.

We found a significant group x time interaction in the rGMV in the right thalamus (x = 6, y = −12, z = −2; p < 0.05, svc, Fig. 2). The estimated values of the right thalamic volumes were significantly increased in the Wo group (Wo: 0.254 ± 0.022 to 0.263 ± 0.023, p < 0.05, paired *t*-test), but not in the Ho group (0.245 ± 0.019 to 0.237 ± 0.029, p = 0.13, paired *t*-test).

No association was detected between the change in the ratio of the D-CAT1 score and right thalamic volume (r = 0.26, p = 0.28).

## Discussion

This was the first study to examine the impacts of hormonal therapy on brain structural changes and cognitive dysfunction in patients with breast cancer. We found the right thalamic volume reduction and attentional dysfunction recovered shortly after the surgery in the Wo group but not in the Ho group. The findings support our hypothesis that thalamic volume reduction and attentional dysfunction would recover in patients without an adjuvant hormonal therapy.

Our findings suggest that hormonal therapy prevents recovery of brain volume reduction and cognitive dysfunction shortly after surgery in patients with breast cancer. Reportedly, cognitive functions were impaired by hormonal therapy^5^,^18^ and improved after the cessation of treatment^19^. Conversely, brain structural changes due to hormonal therapy remained unclear. Since estrogen protects neuronal cells^25^,^26^, hormonal therapy may impair neurons by reducing the neuroprotective effects of estrogen. Based on our findings, even if hormonal therapy does not have direct neurotoxic effects, hormonal therapy prevents recovery of neuronal damage caused by other neurotoxic agents, such as an anesthesia, inflammation and chemotherapy. Therefore, we hypothesized that hormonal therapy is a factor inhibiting recovery from neuronal damage and cognitive dysfunction.

The above mentioned notion is relevant for a pathogenesis of POCD. Manifestation of POCD comprised dysfunction in various cognitive domains^1^ and affected large areas of brain regions^27^. The variation of POCD is apparently associated with multiple risk factors^27^, including age, lower educational level, pre-existing cognitive impairment, long duration of anesthesia, surgery-induced systematic inflammation and complications after surgery^2^,^3^. In line with the multiple risk factors for POCD, hormonal therapy for breast cancer patients can also be considered an additional risk factor for POCD. In the case of hormonal therapy, reduced neuroprotective effects would inhibit a recovery from different forms of neuronal damage, due to various causes. Therefore, we assumed that predicting specific cognitive domains and brain regions impaired by hormonal therapy would be difficult.

In the present study, even if we revealed one aspect of hormonal therapy as a risk factor for cognitive dysfunction, we did not intend to recommend reducing the dose and/or duration of hormonal therapy. Instead, we proposed improving adherence to hormonal therapy medications by an appropriate assessment and rehabilitation of cognitive dysfunction. Regarding hormonal therapy for breast cancer, long-term medication was recommended to achieve a better prognosis^28^,^29^. A 10-year treatment can approximately halve breast cancer mortality, as opposed to discontinuing treatment after 5 years^28^. Therefore, to improve the recurrence and mortality rates, adherence to medications is crucial in hormonal therapy for breast cancer^30^,^31^. Because adherence to medications was significantly reduced in patients with cognitive dysfunction^32^, an appropriate cognitive rehabilitation or training^33^,^34^ could contribute to a better prognosis, by improving adherence. We thought that the effect of cognitive dysfunction on compliance was too subtle to be noticed. Therefore, a better evaluation, and an attempt to treat the cognitive dysfunction, may lead to better treatment.

Our study had several strengths and a limitation. The strength of the current study was controlling other risk factors for cognitive dysfunction, other than hormonal therapy. Additionally, the prospective cohort design and a uniform protocol of MRI scanning were strengths. Accordingly, we successfully detected the recovery of the right thalamic volume reduction and attentional dysfunction shortly after surgery. A limitation of this study included small sample size, specifically in the Wo group, which limited the topics examined. Further studies investigating larger numbers of breast cancer survivors are necessary.

Despite the limitation, the findings support our hypothesis that the thalamic volume reduction and the attentional dysfunction would recover in patients without an adjuvant therapy. In our previous study, we demonstrated that the general anesthesia impacts brain and its functions in cancer survivors^20^. However, most survivors would recover after surgery if not exposed to other risk factors, such as severe surgical trauma, the occurrence of complications, and adjuvant therapies including hormonal therapies. Our findings provide a better understanding of the role of hormonal therapy in relation to cognitive dysfunction in breast cancer survivors.

## Additional Information

**How to cite this article**: Sekiguchi, A. *et al*. Postoperative hormonal therapy prevents recovery of neurological damage after surgery in patients with breast cancer. *Sci. Rep.* **6**, 34671; doi: 10.1038/srep34671 (2016).

## Figures and Tables

**Table 1:** Demographic characteristics of patients.

	Ho; *n *= 13	Wo; *n *= 7	*P*-value
Age (years)	63 ± 8	57 ± 4	0.06
Height (cm)	158 ± 4	156 ± 6	0.48
Weight (kg)	59 ± 10	55 ± 8	0.39
Days after surgery (days)	250 ± 24	250 ± 22	0.98
Education (years)	13 ± 2	14 ± 2	0.41
Alcohol consumption (years × g/week)	571 ± 1140	21 ± 56	0.11
Left-handed (%)	1 (8)	0 (0)	
Adjuvant hormonal therapy			
anastrozole	12		
letrozole	1		

Values are expressed as means ± SD, or numbers (%).

P-values are derived from t-tests for continuous variables.

Abbreviations: Ho, hormonal therapy; Wo, without adjuvant therapy.
